# TGF-β Signaling in Metastatic Colorectal Cancer (mCRC): From Underlying Mechanism to Potential Applications in Clinical Development

**DOI:** 10.3390/ijms232214436

**Published:** 2022-11-20

**Authors:** Xiaoshuang Li, Yanmin Wu, Tian Tian

**Affiliations:** College of Life Science and Bioengineering, Beijing Jiaotong University, Beijing 100044, China

**Keywords:** colorectal cancer, metastasis, TGF-β signaling, targeting therapy, immune-suppressive

## Abstract

Colorectal cancer (CRC) is a serious public health issue, and it has the leading incidence and mortality among malignant tumors worldwide. CRC patients with metastasis in the liver, lung or other distant sites always have poor prognosis. Thus, there is an urgent need to discover the underlying mechanisms of metastatic colorectal cancer (mCRC) and to develop optimal therapy for mCRC. Transforming growth factor-β (TGF-β) signaling plays a significant role in various physiologic and pathologic processes, and aberrant TGF-β signal transduction contributes to mCRC progression. In this review, we summarize the alterations of the TGF-β signaling pathway in mCRC patients, the functional mechanisms of TGF-β signaling, its promotion of epithelial–mesenchymal transition, its facilitation of angiogenesis, its suppression of anti-tumor activity of immune cells in the microenvironment and its contribution to stemness of CRC cells. We also discuss the possible applications of TGF-β signaling in mCRC diagnosis, prognosis and targeted therapies in clinical trials. Hopefully, these research advances in TGF-β signaling in mCRC will improve the development of new strategies that can be combined with molecular targeted therapy, immunotherapy and traditional therapies to achieve better efficacy and benefit mCRC patients in the near future.

## 1. Introduction

Colorectal cancer (CRC) is a type of cancer in which abnormal cells grow out of control in the large intestine. According to global cancer statistics, more than 1.9 million new cases and 935,000 deaths from CRC occurred in 2020. CRC is the third most commonly diagnosed malignancy worldwide and ranks second in cancer-related mortality. In other words, CRC accounts for 10% of all cancer cases and deaths [1]. In the United States, CRC ranked fourth in estimated new cases and second in estimated cancer-related deaths (thus far) in 2022 [2,3]. Due to the prevalence of obesity and lack of exercise in recent decades, the incidence of CRC is on the rise among the entire population in China, and it is currently the fifth leading cause of cancer death there [4]. The accumulation of genetic mutations and environmental risk factors are the main causes of CRC [5].

CRC metastasis is always a thorny problem in clinical situations. At the time of diagnosis, about 20% of CRC patients already have metastasis and 35–45% succumb to recurrence within five years after surgery [6]. The five-year survival rate of stage I-III CRC patients can be as high as 80%, whereas it drops to roughly 13% for patients with stage IV CRC [7]. It is reported that up to 60% of patients with stage IV CRC develop liver metastasis, demonstrating that the liver is the most common site for CRC metastatic spread [8,9]. The lung is the second most common metastatic target organ for CRC. Consequently, although modern surgical techniques and multidisciplinary systematic care have led to significant improvements in survival, long-term remission can only be achieved in 20% of patients with metastasis, and relapse occurred in 60–70% of patients [10,11]. Therefore, there is an urgent need to identify the underlying mechanisms of metastatic colorectal cancer (mCRC) and for new optimal therapeutic strategies for mCRC to be developed.

The transforming growth factor-β (TGF-β) signaling pathway plays a multifaceted role in various biological processes, such as cell growth and differentiation, apoptosis, cell motility, epithelial–mesenchymal transition (EMT), extracellular matrix (ECM) remodeling, angiogenesis and cellular immune responses [12,13]. Therefore, malfunction of the TGF-β signal pathway, either via genetic mutation or misexpression, is associated with many diseases, including cancer, fibrosis, inflammation, cardiovascular diseases, myelodysplastic syndrome, Marfan syndrome, scleroderma, endometriosis and more [14,15,16]. Currently, 33 members in the TGF-β superfamily have been identified in human beings, including three TGF-β isoforms, three activins, nodal, growth and differentiation factor (GDF) and the bone morphogenetic protein (BMP) subfamily, which are involved in various physiologic and pathologic mechanisms [17]. There are three isoforms of TGF-β: TGF-β1, TGF-β2, and TGF-β3. They manifest different expression patterns, bioavailability and physiological functions in organisms, respectively [18]. In addition to these ligands, downstream intracellular effectors, termed SMAD, are a group of proteins that include eight different members in mammalian cells and can transduce extracellular signals to the nucleus [19]. Aberrant signal transduction of TGF-β signaling may lead to a variety of tumors, including esophageal cancer, hepatocellular carcinoma, pancreatic cancer, gastric cancer, CRC, etc. [20]. TGF-β signaling can suppress tumor development by inhibiting cell proliferation and stimulating cell differentiation in the early stages of cancer. However, it induces tumor progression and metastasis in late stages of cancer, which is known as the “TGF-β paradox” [21]. Variation in the TGF-β pathway is also a common event in CRC tumorigenesis and metastasis. When TGF-β or SMAD are mutated, an abnormal TGF-β signaling pathway would contribute to CRC metastasis [22].

The present review mainly focuses on the role of altered TGF-β signaling in mCRC, the mechanisms through which TGF-β affects CRC metastasis and the clinical application of the key components in TGF-β signaling as potential therapeutic targets for mCRC. These research advances will surely shed new light on TGF-β targeting therapy and benefit the mCRC patients in the near future.

## 2. Alterations in TGF-β Signaling Pathway in mCRC

### 2.1. TGF-β Signaling Pathway

Research has shown that TGF-β signaling is transduced from cell membrane surface receptors to the nucleus. TGF-β ligands secreted by cells disperses in the matrix in an inactive form, and it can be activated in an integrin-dependent manner [23]. TGF-β1 and TGF-β3 can be activated by avβ6 or avβ8 integrins while TGF-β2 cannot, which implies a different mechanism for TGF-β2 [24]. There are three receptors in the TGF-β signaling pathway: TGFBR1, TGFBR2 and TGFBR3. The TGF-β ligand first binds to the TGFBR2 and induces the formation of a hetero-tetrameric complex of TGFBR2 and TGFBR1 [25,26]. Subsequently, this complex causes the TGFBR2 kinase domain to phosphorylate TGFBR1 in a region of the juxtamembrane domain that is rich in glycine and serine residues. This then activates TGFBR1 and subsequently phosphorylates SMAD2/3 [27,28]. Following this, phosphorylated SMAD2 and SMAD3 can be assembled into complexes with SMAD4 and then translocated to the nucleus where they can regulate the expression of target genes [28]. SMAD7, a negative regulator of the TGF-β pathway, competes with SMAD2/3 for the catalytic site of TGFBR1 phosphorylation and thereby inhibits the phosphorylation of SMAD2/3 [29]. SMAD proteins can be divided into three categories, including the common-mediator SMAD (Co-SMAD), the receptor-regulated SMAD (R-SMAD) and the inhibitory SMAD (I-SMAD). The Co-SMAD (SMAD4) is the central mediator of the TGF-β signaling pathway. The R-SMAD (SMAD1, -2, -3, -5 and -8/9) can be phosphorylated by activated type I receptor kinases. The I-SMAD (SMAD6/7) can competitively inhibit R-SMAD phosphorylation and thereby antagonize TGF-β signaling [19].

In addition to the canonical SMAD-dependent signaling pathway, there are also several non-canonical pathways within the TGF-β superfamily, such as the Rho-associated kinase (ROCK) pathway, the phosphoinositide 3-kinase (PI3K)/protein kinase B (AKT) and the mitogen-activated protein kinase (MAPK) pathway [30]. These activated non-canonical SMAD pathways also crosstalk with the canonical SMAD pathway.

### 2.2. Aberrant TGF-β Pathway Signals in mCRC

It is generally accepted that the occurrence of cancer is accompanied by the accumulation of gene mutations [31]. Mutations in TGF-β receptors and SMAD proteins occur more frequently in CRC resulting in malignant phenotypes, whereas mutations in TGF-β ligands are relatively rare.

With advances in next-generation sequencing technology, variations in TGF-β signaling in mCRC on different levels have become more accessible for clinical investigators. According to the Ingenuity Pathway Analysis, Wnt, PI3K/AKT and TGF-β/SMAD signaling are the most commonly mutated pathways in colorectal cancer metastasis [32]. Carcinoembryonic antigen (CEA) is widely used as a prognostic clinical marker of metastasis, and the TGF-β signaling pathway is significantly enriched in CEA-induced colorectal liver metastases (CRLM) according to the Kyoto Encyclopedia of Genes and Genomes (KEGG) pathway analysis [33]. In a study utilizing targeted next-generation sequencing (NGS) to assess 128 patients with mCRC, alterations of TGF-β pathways were identified in 17% of the mCRC tissues [34]. In another study involving 579 patients undergoing CRLM resection, 11.2% of patients were found to have TGF-β mutations [35]. Furthermore, aberrant DNA-methylation-regulated genes showed enrichment in TGF-β signaling pathway based on data of DNA methylation (GSE90709, GSE77955) downloaded from the Gene Expression Omnibus database [36].

Although most studies utilize genetic and pharmacological strategies to investigate TGF-β signaling of all three isoforms, these three isoforms actually function through distinct mechanisms. The knockout mice of the three isoforms demonstrated non-overlapping defects: TGF-β1-null mice showed inflammatory disease, TGF-β2-null mice exhibit multiple developmental defects in a wide range of organs, while TGF-β3 knockout led to defective palatogenesis [37,38,39]. TGF-β1 is expressed more abundantly in the tumor microenvironment (TME) in various human tumors than the other two isoforms and contributes to resistance to checkpoint blockade therapy [40]. TGF-β2 was shown to be involved in neutrophil recruitment in an organoid model of mCRC [41]. In addition, TGF-β1 and TGF-β3 are reported to both be activated in stroma cells and to contribute to the prometastatic process in CRC [42].

The receptor of the TGF-β signaling pathway is indispensable and its change can lead to abnormalities of the pathway. Reports indicated that TGFBR1*6A can switch TGF-β1 growth-inhibitory functions into growth-stimulatory functions, which significantly increased the invasion of SW48 and DLD-1 cells compared with transfected TGFBR1*9A cell lines [43]. The germline allele-specific expression (ASE) of TGFBR1 increases CRC risk for the Caucasian-dominated population in the United States [44]. In the majority of microsatellite instability (MSI) CRC tumors, the gene encoding TGFBR2 has a very high frequency of uniquely inactivating mutations. According to public databases, tumors harboring TGFBR2 mutations showed a greater degree of vascular invasion than tumors without such mutations, which contributes to tumor progression in MSI-positive CRC [45]. In addition, frameshift mutations of TGFBR2 were present in three quarters of late-stage MSI CRC, and this mutation might mediate CRC progression from the early to late stage [46]. In an MSI CRC model cell line, inactivating frameshift mutations of TGFBR2 can reprogram the protein content and regulate the cytokine secretion profile. These changes are related to tumor angiogenesis, migration, metastasis and immune escape of recipient cells [47]. In the HCT116-TGFBR2 MSI CRC cell line model system, which reflects the inverse situation of the TGFBR2-deficient MSI CRC, sialylated β1-integrin is significantly decreased, and variant sialylation could affect metastasis and migration of CRC cells [48]. Additionally, in a cohort of 184 CRC patients and 307 healthy volunteers, male CRC patients with TGFΒR2-875A genotypes had a lower risk of CRC progression and metastasis compared with CRC patients with TGFBR2-875G [49].

Because SMAD proteins are key factors in the transduction of the classical TGF-β signaling pathway, alterations in SMAD proteins play a crucial role in late stages of CRC by contributing to migration and metastasis. As reported, CRC patients who lose SMAD activity are more likely to have lymph node metastasis resulting in a poor prognosis [50]. As reported in an analysis of exome capture DNA sequencing from 224 participants, both those with tumors and without, SMAD4 and TGFBR2 are two commonly mutated genes. The mutation frequency of SMAD4 and SMAD2 in non-hypermutated tumors is 10%, while that of TGFBR2 in hypermutated tumors (including MSI-high) is 51% [51]. According to the sequencing analysis of SMAD4, SMAD2 and SMAD3 in a group of 744 primary CRC patients and 36 CRC cell lines, the prevalence of SMAD4, SMAD2 and SMAD3 mutations was found to be 8.6%, 3.4% and 4.3% in sporadic CRC, respectively. In addition, the mutation spectra of SMAD2/3 were highly similar to that of SMAD4, and joint biallelic hits in SMAD2/3 were highly frequent and mutually exclusive to SMAD4 mutation, indicating the crucial roles of these three SMAD proteins in the TGF-β signaling pathway [52].

According to a series of high-throughput analyses, SMAD4 was one of the most commonly mutated genes in mCRC, which will now be further discussed. The results of one study that used targeted NGS sequencing involving 123 non-MSI-high mCRC patients showed a 22.8% mutation frequency of SMAD4 [34]. Similarly, in another study of 32 mCRC patients, the SMAD4 mutation frequency rate was approximately 6% [53]. Similar results were achieved for SMAD4 mutation rates (15% vs. 14%) in primary and metastatic CRCs by comparing genetic profiles [54]. In CRC patients, SMAD4 mutation and deletion detected with NGS were significantly associated with invasive-front pathological markers [55]. In 330 early onset (EO) mCRC patients, SMAD4 was recurrently mutated, resulting in aberrance of the TGF-β pathway in 30% of patients [56]. Using samples obtained from 32 patients, Lopez-Gomez et al. found that SMAD4 expression was at similar levels and was positively associated between the formalin-fixed paraffin-embedded (FFPE) mCRC tumor and their matched liver metastases [57]. Moreover, there is an increased frequency of SMAD4 alterations in ovarian metastases from CRC, suggesting that the oncogenic properties conferred by aberrant TGF-β signaling may contribute to CRC metastasis to the ovaries [58].

SMAD7 is a crucial negative regulator of the TGF-β signaling pathway [59]. Reports indicated that the expression of SMAD7 was remarkably lower in mCRC tissues than in non-tumor tissues [60]. Compared with control mice, mice injected with SMAD7-expressing clones had elevated levels of TGFBR2 expression and TGF-β secretion in liver metastases, which could then lead to phosphorylation and nuclear accumulation of SMAD2. In the nude mouse CRC model, ectopic expression of SMAD7 promoted CRC metastasis to the liver in the splenic injection model [61].

Furthermore, TGF-β mutations always occur concurrently with variations in other signaling pathways, demonstrating that the accumulation of these mutations in mCRC has a synergistic effect on CRC metastasis. For example, KRAS^G12D^ mutation can induce an EMT-like morphology of tumors when combined with mutations in Tgfbr2^-/-^. Moreover, KRAS activation promotes liver metastasis when combined with adenomatous polyposis coli (APC) ^Δ716^ and TGFBR2 mutations [62]. In the colon epithelium of a CRC mouse model, combined inactivation of APC and TGFBR2 promoted development of adenocarcinoma in the proximal colon, and gasdermin C expression was upregulated by TGFBR2 mutation, resulting in increased CRC cells proliferation [63]. Based on the mCRC mouse model that harbored a KRAS^mut^ allele, conditional null alleles of APC and transformation-related protein 53 (Trp53), the TGF-β pathway was a critical mediator of KRAS^mut^-driven invasiveness, as proven by system-level and functional analysis [64]. Fumagalli et al. found that the accumulation of genetic mutations in the Wnt, epidermal growth factor receptor (EGFR), P53 and TGF-β signaling pathways can drive CRC cells to migrate and grow at distant sites in an orthotopic organoid transplantation model and in engineered human colon tumor organoids [65]. In a Chinese CRLM cohort, CRLM patients with differing primary tumor sites had differences in survival rates, which could be driven by combined variations in the TGF-β, PI3K and RAS signaling pathways [66]. Reports showed patients with mutated SMAD4 had shorter progression-free survival (PFS) than patients with wild-type SMAD4 after receiving anti-EGFR therapy, which may imply a synergistic effect of SMAD4 loss and EGFR in mCRC [67].

In addition, some key proteins or factors can act on the TGF-β signaling pathway and affect CRC progression. Compared with a control group, CRC cells overexpressing Tripartite Motif Containing 25 (TRIM25) exhibit a two-fold higher migration rate. TRIM25 also promotes CRC tumor progression in a nude mice xenograft model by positively regulating the TGF-β signaling pathway [68]. The overexpression of Helicase-like Transcription Factor (HLTF) and activation of Slit2/Robo1 signaling can suppress both CRC cell migration and invasion through the TGF-β/SMAD pathway [69,70]. Prolyl 4-Hydroxylase Subunit Alpha 3 (P4HA3) promotes subcutaneous tumorigenesis in nude mice by upregulating the TGF-β/SMAD signaling pathway, and the knockdown of P4HA3 strongly inhibits the proliferation and invasion abilities of CRC cells [71]. ETS homologous factor could activate the canonical TGF-β pathway through directly upregulating TGF-β1 expression at the transcriptional level and could promote CRC cell proliferation and migration in vitro and in vivo [72]. Glypicans 1 (GPC1) knockdown significantly suppressed levels of TGF-β1 and p-SMAD2, resulting in inhibition of the migration of CRC cells [73]. In vivo experiments showed ZIC2, a protein involved in the advancement of many types of tumors, induced TGF-β1 expression and SMAD3 phosphorylation, resulting in CRLM progression [74].

## 3. Mechanism of TGF-β Functions in mCRC

**Figure 1:**
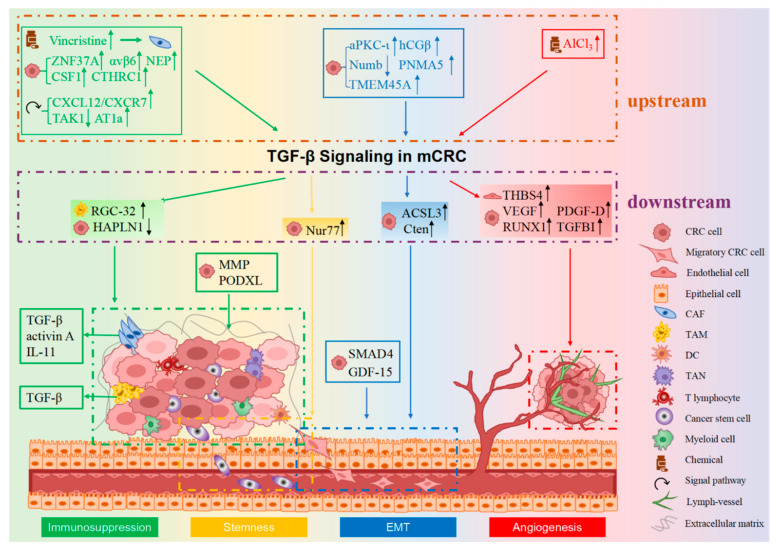
The mechanism of TGF-β signaling in CRC metastasis. TGF-β mainly affects CRC metastasis in four different ways: EMT, angiogenesis, immunosuppression and stemness. Together, they work to facilitate the metastasis of CRC. Tumor cells undergo the EMT process, acquire a mesenchymal-like phenotype in response to TGF-β signaling and then becoming more invasive and spread to distant sites. TGF-β signaling can mediate the formation of new blood vessels, which can promote intravasation of tumor cells from primary lesions into the blood vessels, resulting in tumor metastasis. In the tumor microenvironment (TME), immune cells such as CAFs and TAMs contribute to the immunosuppressive microenvironment and induce dissemination of tumor cells to distant places through TGF-β signaling. Moreover, TGF-β signaling can regulate CSCs in CRC, further promoting tumor metastasis. The molecules upstream or downstream of TGF-β signaling have been enclosed by dotted lines of different colors, and they function through EMT, angiogenesis, immunosuppression and stemness, respectively (distinguished by four different background colors). The symbols in front of the molecules represent whether it is a chemical, a signal factor or a molecule secreted by CRC cells, epithelial cells or immune cells in the TME. EMT, epithelial-to-mesenchymal transition. The legend for different cell types is shown in the lower right.

CRC metastasis is a dynamic, multistep and multifactorial process, which includes the following successive steps: detachment from the primary CRC site, infiltration into adjacent tissues, invasion into blood/lymphatic circulation, transportation through the circulatory system, intravasation from vasculature and formation of CRC colonies in distant sites. Three critical factors contribute to CRC cells migration (pivotal for early metastasis): regulating the EMT process, stemness and the microenvironment of CRC cells. Additionally, angiogenesis facilitates CRC cell transportation to distal locations. TGF-β signaling contributes to mCRC mainly through the following four mechanisms: promoting EMT, facilitating angiogenesis, creating an immunosuppressive microenvironment and regulating the stemness of mCRC (as shown in Figure 1) [21,28].

### 3.1. TGF-β Signaling in EMT in mCRC

Epithelial cells undergoing EMT will lose their apicobasal polarity and adhesion, acquire motile mesenchymal characteristics and become more invasive, which contributes to the onset of CRC metastasis [75]. Epithelial and mesenchymal cells can be distinguished by specific molecular markers expressed in cells. For instance, epithelial cells express E-cadherin and cytokeratins, while N-cadherin, Snail, Slug and Vimentin are markers of mesenchymal cells [76]. Cells undergoing the EMT process are distinguished by the loss of E-cadherin expression, a decrease of epithelial cell junctions and cytoskeleton and display a mesenchymal pattern with enhanced cell motility and invasiveness [77].

TGF-β signaling is an essential regulator of the process of EMT. As reported, TGF-β can induce the EMT process by downregulating the expression of tight junction proteins, resulting in weakened tight junctions, which is the key point for EMT induction of TGF-β signaling [78]. SMAD4 is demonstrated to downregulate the expression of Claudin 1, which contributes to CRC metastasis [79]. Most of these observations were made in vitro, although the results of in vivo experiments are more convincing and important. In light of research in human SW480 CRC cells, TGF-β1 can induce Alu RNA expression, the accumulation of which promotes the EMT process, and Alu expression significantly correlates with CRC progression [80]. TGF-β1 upregulates the expression of C-terminal tensin-like (Cten) and EMT markers, and it promotes the cell motility of the CRC cell lines SW620 and HCT116 [81]. TGF-β1 can also induce the upregulation of acyl-CoA synthetases 3 (ACSL3) which produces ATP and reduces NADPH, thus sustaining redox homeostasis and mediating the EMT and metastasis of CRC cells [82]. A functional study indicated that TGF-β can induce SMAD4-dependent EMT followed by apoptosis in HCT-116 and DLD1 CRC cell lines [83]. As reported in CRC cell assays and murine models, acidosis-induced TGF-β2 activation promotes the formation of lipid droplets, which provides energy for cancer cell metastasis and partially promotes EMT [84]. SMAD4 in TGF-β signaling is frequently inactivated in human CRC, and SMAD4 codes for a transcription factor central to canonical TGF-β signaling. Therefore, it is generally understood that EMT will not occur in SMAD4-mutant tumors. However, in SMAD4-mutant CRC cell lines and analyses of human CRC transcriptomes, EMT is not categorically precluded. Possible explanations for this may be that SMAD4-mutant tumors escape the tumor-suppressive function of TGF-β or undergo SMAD4-independent EMT [85]. Moreover, CRC patient tissues exhibited higher GDF-15 expression compared with non-cancerous controls, and in the human CRC cell line LoVo, the overexpression of GDF-15 could upregulate the marker genes of mesenchymal cells. Thus, GDF-15 could lead to EMT and promote CRC cell invasion and migration [86]. Based on the systematic analysis of samples from seven CRC patients, it was found that some potential EMT biomarkers were enriched in TGF-β/Snail and TNF-α/nuclear factor-κB (NF-κB) pathways, and the integrated pathway may be the main axis connecting cancer cells with their TME during EMT [87]. In an immunohistochemical study of 48 resected CRC specimens, SMAD4 was positively linked with the expression of Snail-1, Slug and Twist-1, while it was negatively correlated with E-cadherin expression, implying that SMAD4 promotes the process of EMT [88].

There are also other factors that affect EMT by regulating the TGF-β pathway. For example, atypical protein kinase C-ι (aPKC-ι) knockdown inhibits TGF-β1-induced EMT and cell migration in CRC cells [89]. Furthermore, in 5-fluorouracil (5-FU)-resistant CRC cell lines, knockdown of transmembrane protein 45A (TMEM45A) attenuated multidrug-resistance-enhanced EMT by suppressing the TGF-β/SMAD signaling pathway [90]. In studies utilizing cell line experiments and nude mouse models, Numb expression was negatively correlated with TNM stage and lymph node metastasis, and inhibiting Numb expression promoted the EMT process and the invasion of CRC cells induced by TGF-β [91]. It was found that Paraneoplastic antigen Ma family number 5 (PNMA5) accelerated CRC cell proliferation, invasion and migration in nude mice lung metastasis models, and the knockdown of PNMA5 attenuated TGF-β-induced EMT in CRC cells [92]. As reported in cell assays and mouse xenograft tumors, beta human chorionic gonadotropin (hCGβ) changed expression of EMT-associated genes, and these changes could be reversed by TGFBR1 and TGFBR2 inhibitors, indicating that hCGβ induces EMT in a manner that depends on the TGF-β pathway [93].

### 3.2. TGF-β Signaling in Angiogenesis in mCRC

Angiogenesis in the TME is a pivotal process that promotes tumor development and metastasis [94]. Newly formed blood vessels can provide oxygen and nutrients to tumor cells as well as allow them to enter into blood circulation and metastasize to distant sites [95]. 

First, the TGF-β pathway can regulate tumor metastasis by affecting vascular endothelial growth factor (VEGF). In the CRC HCT116 cell line, the upregulation of VEGF expression caused by the absence of SMAD4 enhanced vascular density and promoted the development of metastasis [96]. Additionally, SMAD4 overexpression can inhibit CRC growth by inhibiting VEGF-A and VEGF-C expression in the HCT116 cell line and an promote tumor cell apoptosis in HCT116 cells and nude mouse models [97]. There are primarily two histopathological patterns of vascular changes in CRLM: angiogenic desmoplastic and non-angiogenic replacement [98]. Overexpression of Runt-Related Transcription Factor-1 (RUNX1) in cancer cells of the replacement lesions, which is mediated by TGF-β1 and thrombospondin 1 (TSP1), enhances cell motility to achieve vessel co-option [98]. TGF-β expression is increased in the AlCl_3_-exposed human CRC cell line HT-29, and this particularly promoted endothelial cell angiogenesis via the induction of VEGF secretion [99]. In an orthotopic mouse model of liver metastasis, the inhibition of TGF-β-induced protein ig-h3 (TGFBI) suppressed angiogenesis of CRC cells and inhibited the progression of CRLM [100]. Second, the synergistic effects of TGF-β and other signal cascades can stimulate angiogenesis by accelerating endothelial cell migration and proliferation [77]. TGF-β can interact with other proteins or pathways to foster angiogenesis in mCRC. The downregulation of platelet-derived growth factor-D (PDGF-D), a downstream signal of TGF-β, inhibited the growth, migration and angiogenesis of CRC cells in vitro and in vivo [101]. Thrombospondin-4 (THBS4), an ECM protein, plays an essential role in the TME and augments the effects of TGF-β1 on angiogenesis [102,103].

### 3.3. TGF-β Signaling in Immunosuppressive Microenvironment in mCRC

Growing evidence has shown that the TME performs a significant role in tumor initiation, progression and metastasis. The TME comprises non-cancerous cells in the tumor, including cancer-associated fibroblasts (CAFs), endothelial cells, pericytes and different types of immune cells (dendritic cells (DCs), tumor-associated macrophages (TAMs), tumor-associated neutrophils (TANs), natural killer (NK) cells, myeloid cells, T cells, B cells, monocytes etc.), as well as non-cellular components, including ECM and soluble products such as collagen, various cytokines, chemokines and other factors that contribute to CRC metastasis [104,105,106]. Direct cell-to-cell contact between cancer cells and secretion of cytokines in the TME caused crosstalk, resulting in CRC progression and ultimately metastasis. It was reported that the activity of TGF-β signaling in TME cells such as T cells, macrophages, endothelial cells and fibroblasts improved the organ colonization efficiency of CRC cells, while treating the mice with the TGFBR1-spesific inhibitor LY2157299 inhibited CRC metastasis formation [42]. Elevated TGF-β expression levels is an important feature in the TME of CRC, and TGF-β signaling can regulate the development of CRC, form the system structure of tumors and inhibit the activity of anti-tumor immune cells, which results in an immunosuppressive microenvironment [28,107,108].

Here we summarize recent research and find that most of studies focused on CAFs and immune cells such as TAMs, TANs, DCs, T cells, myeloid cells and monocytes. Only a few studies on TGF-β signaling-mediated CRC progression and metastasis were related to collagen (discussed in the CAF section). TGF-β-signaling-related CRC metastasis involving CAFs and immune cells will be further discussed in detail in the following sections.

#### 3.3.1. CAFs

CAFs are the most numerous cells in the TME, and they affect CRC metastasis by regulating TGF-β signaling directly or indirectly [108,109]. TGF-β is mainly produced by CAFs in CRC, and increased TGF-β promotes T cell exclusion and inhibits the effector phenotype acquisition of type 1 T helper cells (TH1). It has been reported that inhibition of TGF-β enhances the cytotoxic T cell response to tumor cells, thus suppressing liver metastasis [110]. TGF-β activates CAFs to secrete activin A, a TGF-β family member, which induces colon epithelial cell migration and EMT, resulting in a more metastatic phenotype of CRC [111]. Wang et al. have recently reported that the activation of C-X-C motif chemokine ligand 12 (CXCL12)/CXCR7 axis drove CRC cells to secrete exosomal miR-146a-5p and miR-155-5p, which could be taken up by CAFs, thus enhancing CAF activation via JAK2-STAT3/NF-κB signaling. CAFs could secrete more inflammatory cytokines, including TGF-β, further promoting EMT and CRC metastasis to the lung in vivo [112]. ZNF37A, which is upregulated in CRC, is reported to facilitate tumor cell metastasis to the lung and liver via the activation of Thrombospondin Type-1 Domain-Containing protein 4 (THSD4)/TGF-β signaling, and increased TGF-β secretion contributes to transforming fibroblasts to CAFs in the TME, further promoting CRC metastasis [113]. Integrin αvβ6 secreted by CRC cells induced the expression of TGF-β, thereby converting fibroblasts into CAFs and promoting CRC metastasis through the stromal cell derived factor-1 (SDF-1)/C-X-C motif chemokine receptor type 4 (CXCR4) axis [114]. Treatment of co-cultured CRC and CAF-like cells with vincristine, which is a chemotherapy drug used widely in mCRC clinical treatment, increased the secretion of TGF-βs, induced EMT and promoted the formation of CAFs, thereby enhancing the invasion and metastasis of CRC [115]. Interleukin-11 (IL-11) secreted by TGF-β-stimulated CAFs is a TGF-β target gene, and it activated GP130/signal transducer and activator of transcription 3 (STAT3) signaling in CRC cells and promoted the initiation of CRC cells to metastasis [42]. Endoglin, a TGF-β family coreceptor produced by CAFs, enhanced CRC cell metastasis to the liver in both zebrafish and mouse models [116]. In addition, it has been demonstrated that tumor necrosis factor-related apoptosis-inducing ligand (TRAIL) secreted by SMAD4-deficient CRC cells promotes fibroblasts to produce BMP2, resulting in CRC cell invasion and metastasis [117].

Moreover, TGF-β1 can be secreted by tumor cells in metastasis. Neutral endopeptidase (NEP) co-culturing human colon cancer cell line SW620 (derived from metastatic tumors) with normal colon fibroblasts induced a significant increase in expression of TGF-β1 in SW620 cells, and this effect could be reversed by deletion of NEP [118]. As reported, TGF-β1 promoted the co-migration of colon cancer cells and CAFs, resulting in enhanced liver metastasis and tumor burden [119]. CAF-derived exosomal microRNA (miR)-17-5p caused CRC cells to secrete TGF-β1 into the TME through RUNX3/MYC/TGF-β1 signaling, which triggered CAFs to release more exosomal miR-17-5p to CRC cells, thus establishing a positive feedback loop for CRC metastasis [120]. In CRC, fibroblasts could be converted to CAFs via IL-1β/TGF-β1 signaling, and both TGF-β-activated kinase 1 (TAK1) and TGFBR1 inhibitors suppressed CRC metastasis and CAF accumulation [121]. Two additional studies revealed that CXCR4/TGF-β1 signaling plays an important role in the transformation of mesenchymal stem cells or hepatic stellate cells into CAFs, further promoting CRLM [122,123]. 

However, CAFs can also suppress CRC progression in some situations. In a genetically modified metastatic CRC mouse model, depletion of alpha smooth muscle actin (αSMA)^+^ CAFs resulted in an increase of forkhead box protein 3 (Foxp3)^+^ regulatory T cells (Tregs) and suppression of CD8^+^ T cells via BMP4/TGF-β1 paracrine signaling, ultimately promoting CRC invasiveness and lymph node metastasis [124]. A recent study showed that gremlin 1 (GREM1) and the immunoglobulin superfamily contain leucine-rich repeat (ISLR), representing two different types of fibroblast subpopulations that exert opposing roles in the signal transduction of BMP. Neutralization of GREM1 or overexpression of ISLR in fibroblasts could reduce CRC hepatic metastasis [125].

Furthermore, decreased expression of hyaluronan and proteoglycan link protein-1 (HAPLN1) regulated collagen deposition in CRC via the TGF-β signaling pathway, and increased collagen resulted in TME changes and CRC cell proliferation, migration and invasion [126].

#### 3.3.2. Immune Cells

TAMs, one of the most common immune cells in the TME, have been reported as key contributors to promote tumor metastasis [127,128]. Liu et al. found severe TAM infiltration in tumor tissues of mCRC patients, and TAM-derived TGF-β could activate HIF1α/TRIB3/β-catenin/Wnt signaling to enhance CRC progression [129]. GDF-15, secreted by macrophages, is a divergent member of the human TGF-β superfamily, and it can increase expression of EMT genes, thereby promoting the invasion and metastasis of CRC via the ERK1/2/c-Fos signaling pathway [130]. Shimizu et al. found that Kupffer cells, known to be resident hepatic macrophages, released TGF-β1 and promoted liver metastasis of CRC through angiotensin II subtype receptor 1a (AT1a) signaling. Moreover, depletion of Kupffer cells reduced metastatic areas [131]. It has been proven that TGF-β1 secretion of CRC cells upregulated macrophage expression of Response Gene to Complement 32 (RGC-32) and thus enhanced macrophage migration and promoted tumor progression [132]. Recently, Chen et al. found that oxaliplatin-based chemotherapy induced TAM recruitment to release TGF-β, which was mediated by CRC-cell-derived CSF1, resulting in programmed cell death-Ligand 1 (PD-L1) upregulation and an immunosuppressive TME. Inhibition of PD-L1 expression in CRC could make cancer cells sensitive to chemotherapy, reduce CRC lung metastasis and increase infiltration of CD8^+^ T cells. Both CSF1R^+^ TAM depletion and TGF-β receptor blockade combined with chemotherapy could inhibit tumor growth significantly [133]. Through specific differentiation, macrophages can be polarized into two different phenotypes: activated M1-type and alternatively activated M2-type. M1-type macrophages inhibit tumor growth and progression, whereas M2-type macrophages induce the progression and metastasis of tumors in CRC [127,134]. Ma et al. found that M2-type macrophages were positively correlated with infiltrating Foxp3^+^ Tregs in CRC, which may promote the development of CRC via the TGF-β/SMAD signaling pathway [135]. Cai et al. reported that M2-type macrophages that secreted TGF-β promoted EMT by activating the SMAD2,3-4/Snail/E-cadherin signaling pathway, resulting in CRC lung metastasis [136]. Zhang et al. revealed that Collagen Triple Helix Repeat Containing 1 (CTHRC1) secreted by CRC cells induced macrophages to the M2-type through activation of TGF-β signaling, further enhancing CRC liver metastasis [137]. Recently, Li et al. developed a thermosensitive hydrogel called Gel/(regorafenib + NG/LY3200882 (LY)), which could sequentially release regorafenib and LY (a selective TGF-β inhibitor) in tumor cells. Using colorectal tumor-bearing mouse models, they found that Gel/(regorafenib + NG/LY) can effectively inhibit tumor growth and liver metastasis, which was achieved by increasing levels of CD8^+^ T cells, reducing infiltration of TAMs and myeloid-derived suppressor cells and shifting macrophage polarization from M2-type to M1-type in TME [138].

Apart from CAFs and TAMs, other cellular components, such as TANs, myeloid cells, monocytes, DCs and T cells, in the TME can also affect CRC metastasis through TGF-β signaling. TAN infiltration was demonstrated to be positively correlated with the clinical stage of CRC patients [139]. Anti-TGF-β treatment attenuated tumor growth, which was mediated by inhibition of PI3K/AKT signaling pathways in TANs and TGF-β/SMAD signaling pathways in CRC cells [140]. Activation of epithelial NOTCH1 enhanced epithelial TGF-β2 expression and facilitated liver metastasis of CRC through TAN infiltration, which was mediated by TGF-β signaling. Neutrophil depletion led to increased CD8^+^ T cells in both primary tumors and livers and decreased metastasis. In addition, blocking TGF-β signaling in neutrophils can effectively reduce CRC metastasis [41]. Using mouse xenograft models, Itatani et al. found that a deficiency of SMAD4 in human CRC cells upregulated CCL15 expression, thus recruiting CCR1^+^ myeloid cells and promoting liver metastasis of CRC [141]. Furthermore, inflammation is an important driver for CRC development and metastasis. CRC cells treated with lipopolysaccharide-stimulated monocyte conditioned medium showed reduced expression of Growth Factor Independence 1 and enhanced EMT and CRC cell metastatic formation, which might have been mediated by TGF-β signaling [142]. Wang et al. suggested that silencing poly (ADP-ribose) glycohydrolase (PARG) in CT26 cells could suppress liver metastasis of colon carcinoma by suppression of poly (ADP-ribose) polymerase (PARP) and NF-κB and that it could reduce secretion of IL-10 and TGF-β, thus promoting the proliferation and differentiation of DCs and T cells, resulting in inhibition of metastasis by changes in immune function [143]. Treg and T helper 17 (Th17)-related genes seem to contribute greatly to CRC development and progression. Miteva et al. investigated the expression of Treg and Th17-related genes in CRC tissues and found that Foxp3, IL-10 and TGF-β1 expression was increased in CRC metastases in contrast to IL17A and NOS2. Treg and Th17-related gene expression in both primary tumor and regional lymph nodes might provide a suitable microenvironment for accelerating CRC metastasis [144]. The mechanism by which other cells in the TME influence T cells via TGF-β signaling directly or indirectly was covered in the previous section [41,110,124,133,135,138,143].

### 3.4. TGF-β Signaling in Stemness in mCRC

Most tumors, including CRC, contain a small population of cancer stem cells (CSCs) which are regarded as key contributors to tumor generation, progression, recurrence, metastasis and chemotherapy drug resistance [145,146]. According to recent studies, the TGF-β signaling pathway can affect metastasis of CRC by affecting CSCs in CRC or the stemness of CRC cells. 

Mesenchymal stem cells co-cultured with CRC cells showed enhanced invasive ability, which was mediated by increased expression of TGF-β1 and decreased expression of p53, resulting in effective inhibition of CRC metastasis [147]. Reports suggested that TGF-β could convert Nur77’s role from cancer inhibition to cancer promotion, which is associated with CRC stemness, metastasis and oxaliplatin resistance [148]. CSCs have specific markers on their surface. CD51, a novel functional marker for colorectal CSCs, could increase the sphere-forming abilities, tumorigenic capacities and migratory potentials of CRC cells, and it may regulate EMT and chemoresistance through TGF-β/SMAD signaling [149]. In a novel mouse model of CRLM, proteomic analysis revealed that the expression of CRC stem cell markers in CRC cells was elevated compared with the non-metastatic model, and the expression of these markers was regulated negatively by the TGF-β/SMAD4 pathways [150].

### 3.5. Other Mechanisms of TGF-β in mCRC

In addition to the four mechanisms mentioned above, TGF-β can also affect the metastasis of CRC through some additional mechanisms. TGF-β regulates matrix metalloproteinase (MMP) expression in cancer cells, while MMPs produced by either cancer cells or stroma cells activate latent TGF-β, together facilitating progression of CRC [151]. The expression of TGF-β and the podocalyxin-like (PODXL) protein in CRC cells could increase under radiation and then promote ECM deposition, resulting in cell migration and invasiveness [152]. Bioinformatic analysis and functional characterization indicated that TGF-β and Snail promoted CRC migration by preventing degradation of the non-coding RNA LOC113230-related argininosuccinate synthase 1 (ASS1) [153]. Reports indicate that cancer epithelial cells show a robust outward apical pole throughout the process of dissemination, which is referred to as tumor spheres with inverted polarity (TSIPs). TSIPs form and propagate via the collective apical budding of hypermethylated CRCs downstream of TGF-β signaling, which could drive the formation of peritoneal metastases [154]. Moreover, TrkC, which was overexpressed in CRC, could also increase the ability to form tumor spheroids, thus enhancing the metastatic potential of CRC by activation of AKT and suppression of TGF-β signaling [155]. It has been demonstrated that TGF-β inhibits lymph angiogenesis by inhibiting collagen and calcium-binding EGF domain-1 (CCBE1) expression, and CCBE1 has a pro-tumorigenic role in lymphatic metastasis of CRC [156]. TGF-β2 could enhance the metastatic potential of human CRC cell lines via upregulating the expression of catalase and controlling H_2_O_2_ output [157].

## 4. Potential Application of TGF-β Signaling in Diagnosis and Prognosis of mCRC 

Specific molecular biomarkers are significant tools for early diagnosis and prognosis of mCRC, and early prognosis is the most successful and effective method to improve the survival rate of CRC patients [158]. Through integrative clustering of the expression profiles of miRNA-correlated genes and methylation-correlated genes, four molecular subtypes (S-I, S-II, S-III and S-IV) were confirmed in CRC patients from The Cancer Genome Atlas (TCGA) [159].

Due to the poor prognosis of patients with mCRC, it is crucial to make a rapid and accurate diagnosis of mCRC based on specific biomarkers as early as possible. Many reports on applications of TGF-β signaling in the diagnosis of mCRC have been published. According to relative mRNA quantification, the expression of TGF-β1 in CRC distant metastases is significantly increased compared with primary tumor tissues [160]. The increased expression of TGF-β2 is a dependable predictor of lymph node metastasis in CRC patients [161]. Compared with healthy controls, serum levels of GDF-15, a member of the TGF-β superfamily, were remarkably upregulated in mCRC patients and had the same sensitivity as the standard tumor marker CEA, indicating that GDF-15 could be an effective biomarker in mCRC patients [162]. The morphogen nodal is dramatically overexpressed in malignant transformation in CRC, and it could be a potential marker for the consensus molecular subtype 4 (CMS4) subtype of CRC [163].

More reports of TGF-β signaling on prognosis have been shown in mCRC patients. The combined activin and TGF-β ligand expression score was utilized to predict shorter OS in a group of 40 CRC tumors, 10 metastasis and 10 control samples [164]. In EO mCRC patients, mutated TGF-β pathways were found to be associated with unfavorable OS by capture-based targeted sequencing [56]. For the TGFΒ1-509C/T single nucleotide polymorphism (SNP), CRC patients with the TT allele have the shortest median survival, which is due to malignant progression in advanced stages [165]. High levels of TGF-β in blood samples are negatively correlated with PFS in mCRC patients before treatment with regorafenib, and these results suggest that the cytokine signature can distinguish whether patients respond to regorafenib treatment or not [166]. The survival analysis demonstrated that MYC and TGF-β pathway alterations were related to a shorter OS in mCRC patients, and this negative prognostic impact was maintained after receiving an anti-EGFR antibody [34]. Using tumor tissues from 230 mCRC patients treated with oxaliplatin combined with 5-FU chemotherapy, Baraniskin et al. reported that SMAD4 expression was decreased in 34% of mCRC samples, and these patients had a shorter PFS and OS compared with patients in which SMAD4 is stably expressed [167]. In an analysis of multiple gene mutation assessments in 123 regorafenib-treated mCRC patients, researchers detected a SMAD4 mutation in one patient who had long response to regorafenib [168]. In addition, SMAD4-mutated patients performed significantly worse in terms of PFS than those without SMAD4 mutations in a study with 76 regorafenib-treated mCRC patients [169]. Whole exome sequencing analysis of 77 mCRC patients revealed that SMAD4 mutations were significantly correlated with poor prognosis [170]. Patients with SMAD4 mutations developed CRLM and had worse OS after hepatic resection [171]. In general, changes in TGF-β signaling pathways in CRC cause cancer cells to become more aggressive and more likely to metastasize; thus, patients harboring mutations in TGF-β signaling components often have a poor prognosis.

Other members associated with the TGF-β family can also be utilized as prognostic biomarkers in mCRC. Studies imply that high tumor expression of activin A (a homodimer of inhibin beta A) is associated with poor prognosis in patients with CRC, and activin A receptor type 2A (ACVR2A) (a membrane receptor in the TGF-β signaling pathway) depletion plays an important role in CRC distant metastasis and may be recommended as a prognostic biomarker in CRC patients [172,173]. As mentioned in the previous section, the entry level of GDF-15 might be a prognostic factor that is strongly relevant to OS in mCRC patients [162]. Overexpression of inhibin subunit beta B (INHBB) (a protein-coding gene that participates in the synthesis of TGF-β family members) was positively associated with CRC invasion and distant metastasis, suggesting that it could be a potential prognostic biomarker for mCRC [174]. Furthermore, knockdown of high inhibin, beta A (INHBA) in vitro can inhibit CRC cell migration and invasion by inhibiting the TGF-β pathway, and INHBA expression is closely related to poor prognosis in CRC patients [175]. Overexpression of lncRNA-activated by TGF-β (lncRNA-ATB) was significantly associated with CRC metastasis, and lncRNA-ATB expression could be a prognosis biomarker of OS in CRC patients [176]. Additionally, TGFBR2 deficiency is positively correlated with upregulation of miR-31-3p [177], which is a predictive biomarker for the efficacy of anti-EGFR treatment that mCRC patients received [178].

## 5. Targeting TGF-β Signaling Pathway in mCRC

As we have discussed in previous sections, TGF-β signaling plays a significant role in CRC metastasis by promoting EMT, facilitating angiogenesis, contributing to an immunosuppressive TME, regulating stemness of mCRC cells and other mechanisms. These research achievements led us to explore more strategies targeting TGF-β signaling which may have promising application prospects in mCRC therapy. So far, long non-coding RNAs (lncRNAs), miRNAs, kinase inhibitors and natural compounds are common strategies that have been utilized for TGF-β targeting in clinical trials, and new sequencing techniques could facilitate the development of personalized medicine for mCRC patients. These various factors targeting TGF-β signaling in CRC metastasis are summarized in Table 1.

### 5.1. LncRNAs

LncRNAs are a class of multifunctional noncoding RNAs whose sizes are greater than 200 nucleotides. Many recent studies have shown that lncRNAs play a critical role in regulating progression and metastasis in CRC [179,180]. In CRC patients, outlier expression of the lncRNA MIR31HG was observed and was characterized by elevated EMT, TGF-β and IFN-α/γ gene expression signatures in pre-clinical models [181]. The lncRNA CTBP1-AS2 increased CRC cell invasion and decreased apoptosis by activating the TGF-β/SMAD2/3 pathway and was closely associated with worse survival rate in CRC patients [182]. Additionally, the lncRNAs TP73-AS1 and MIR503HG inhibited the migration and invasion of CRC cells by inactivating TGF-β1 and downregulating TGF-β2, respectively [183,184]. Transwell assays showed that TGF-β2 overexpression increased cell invasion, while overexpression of the lncRNA HOXC-AS3 could reverse the effect of overexpression of TGF-β2 [185]. According to an experiment in CRC cell lines, silencing of the lncRNA ezrin antisense RNA 1 (lncRNA EZR-AS1) accelerated CRC cell apoptosis and inhibited the migration and EMT of CRC cells by blocking TGF-β signaling [186]. Silencing of the lncRNA MIR22HG promoted CRC cell proliferation and tumor metastasis in vitro and in vivo by competitively interacting with SMAD2 [187]. Moreover, LINC00941 enhanced invasive capacity and accelerated lung metastasis by activating EMT by directly binding SMAD4 and preventing SMAD4 protein degradation in mCRC [188].

### 5.2. MiRNAs

Increasing evidence has shown that miRNAs that regulate TGF-β signals have significant roles in the progression and metastasis of CRC; they act as oncogenes or tumor suppressors to regulate expression of specific targets [189]. Compared with primary CRC, a series of studies on related miRNAs reported epigenetic alternations in CRLM [36]. MiR-425 and miR-576 were significantly upregulated in CRLM based on GSE81581 and GSE44121 datasets, and the two miRNAs were associated with CRC metastasis by co-participating in inhibition of the TGF-β signaling pathway [190]. It has been proven that upregulation of miR-329 suppresses CRC cell invasion by inhibiting TGF-β1, and low expression of miR-329 is correlated with lymph node metastasis in CRC patients [191]. Upregulated expression of plasma miR-211 and 25, which are relevant to the high expression of TGF-β1 in CRC patients, was positively correlated with lymph node metastasis [192]. In HCT116 colon cancer cells in which kallikrein 6 was knocked down, miR-203 was demonstrated to inhibit migration and invasion of CRC cells by inhibiting the EMT through suppression of TGF-β2 [193].

Some miRNAs function through targeting the TGF-β receptors in mCRC. For instance, downregulation of miR-301a was shown to inhibit CRC migration and invasion both in vitro and in vivo by repressing TGFBR2 protein expression in an analysis containing 48 cases of CRC tissues, adjacent non-tumor tissues and five CRC cell lines [194]. TGFBR2 repression by overexpression of the entire miR-371~373 cluster decreased tumor-initiating potential in tumor-initiating cells [195]. Reports indicated that miR-3191 promoted CRC cells migration and invasion by downregulating TGFBR2 [196]. Artificial overexpression of miR-490-3p inhibited cell migration and invasion in CRC cell lines through the suppression of TGFBR1 and MMP2/9 [197]. CircFAM120B overexpression blocked CRC cell migration and reduced the expression of miR-645. In addition, TGFBR2 was a target of miR-645, whose inhibition suppressed CRC cell migration and can be restored by TGFBR2 knockdown [198]. As reported in the LoVo cell experiment and subcutaneous tumor model, the inhibition of miR-424 suppressed migration and invasion of CRC cells as well as arrested CRC cells at the G0/G1 phase by repressing TGFBR3 [199].

There are also many reports on miRNAs that affect CRC metastasis through SMAD proteins in the TGF-β signaling pathway. Functional studies showed that miR-27a inhibited SMAD2 expression at transcriptional and translational levels and that it promoted colon cancer cell apoptosis and attenuated cell migration [200]. Ectopic expression of miR-140 inhibited EMT partially through downregulating SMAD3, and it enhanced invasive capacities of CRC cells in vitro, while overexpression of miR-140 inhibited the metastasis of CRC in vivo [201]. Studies confirm that miR-20a-5p promoted the invasion and metastasis ability of CRC cells and liver metastasis, as well as accelerated the EMT process by reducing SMAD4 expression, which is slightly controversial compared with most other reports [202]. Furthermore, bioinformatic predictions and experimental validation demonstrated that SMAD7 is a direct target of miR-25 in mCRC, and miR-25 inhibition could promote the migratory ability of CRC cells via the suppression of SMAD7 [203]. High miR-4775 expression promoted CRC cell metastasis and EMT via downregulating SMAD7 and thereby activated the TGF-β pathway both in vitro and in vivo [204]. Wang et al. demonstrated that miR-21-mediated inhibition of SMAD7 accelerated TGF-β-dependent EMT in CRC, indicating that loss or inhibition of SMAD7 could promote CRC metastasis [205]. This implies that the overexpression of circTBL1XR1 enhances the proliferation and migration of CRC cells by binding to miR-424, which inhibits SMAD7 [206]. All these examples show that miRNAs can inhibit SMAD7, promote TGF-β-dependent EMT and contribute to CRC metastasis.

### 5.3. Kinase Inhibitors

Some kinase inhibitors for the TGF-β signaling pathway have been evaluated in various models for mCRC combination treatment to improve the efficacy of therapy. TGFBR1, TGFBR2 and TGFBR3 mutations were found in mCRC patients who responded to regorafenib, suggesting that the TGF-β signaling pathway may play a leading role in the regorafenib response [207]. While using regorafenib, a novel oral multikinase inhibitor, mCRC patients with SMAD4 mutations or activation of the TGF-β pathway showed a worse PFS, which was demonstrated by NGS-based cancer panel tests [169]. Based on a TGF-β-inducible reporter system, Zhang et, al. showed that the TGF-β receptor kinase inhibitor LY2109761 inhibited CRLM by blocking the tumor promoting function of TGF-β in vivo [208]. In a colon cancer liver metastases murine model, mice were treated with adoptive natural killer cells combined with the TGF-β receptor kinase inhibitor LY2157299, and a significant eradication of liver metastases occurred [209]. A study using human CRC cell lines demonstrated that sitagliptin can inhibit CRC cell metastasis by partially blocking TGF-β1-driven EMT [210]. According to the targeted NGS analysis of tumor samples with pre- and post-cetuximab treatment, the copy number of the SMAD4 gene changed, while the TGF-β signaling pathway had various recurrent mutations [211]. The therapeutic potential of these variants requires further clarification.

Correspondingly, dual treatments with the TGF-β galunisertib (LY2157299) inhibitor and AXL inhibitor prominently reduced migration capabilities of human CRC cell lines [212]. Moreover, melatonin, hyperbaric oxygen and combined treatments inhibited CRC metastasis through a variety of mechanisms, including restraining cancer stemness [213]. However, the application of inhibitors should be taken under careful consideration, as it has been reported that epithelial truncation of TGFBR2 leads to fatal inflammatory diseases and invasive CRC in APC mice (a model of intestinal neoplastic disease). Moreover, APC mice with global suppression of TGF-β signaling present with an overall increase in inflammation and tumor formation, suggesting that CRC patients treated with TGF-β inhibitors may have a worse outcome by enhancing inflammatory responses [214].

### 5.4. Natural Compounds and Chinese Herbal Formulas

Some natural compounds and Chinese herbal formulas can also be utilized as indirect approaches for targeting TGF-β. In vitro results from transwell and scratch wound assays demonstrated that solasodine inhibited CRC cell invasion and migration, which was strengthened by TGF-β1. Solasodine also attenuated TGF-β1-induced EMT in vivo [215]. A traditional Chinese herbal medicine, Hedyotis diffusa Willd, may develop its anti-metastatic activity by restraining TGF-β/SMAD4 pathway-mediated EMT in 5-FU-resistant CRC cells [216]. In addition, baicalin caused cell cycle arrest in the G1 phase and EMT inhibition through inhibiting the TGF-β/SMAD pathway in CRC RKO and HCT116 cell lines [217]. Celastrol significantly inhibited human CRC cells growth, adhesion and metastasis by repressing the TGF-β1/SMAD signaling pathway [218]. The ethanol extract of *Scutellaria barbata* D. Don (EESB) significantly reduced the migration ability of HCT-8 cells in a dose-dependent manner. Furthermore, EESB decreased expression of MMPs and proteins involved in PI3K/AKT and TGF-β/SMAD signaling [219]. Mechanistically, metformin can block the activation of TGF-β signaling by INHBA, which is an important ligand of TGF-β signaling. It can then downregulate the activity of the PI3K/AKT pathway, leading to cell cycle arrest and inhibition of the proliferation of CRC [220]. In vitro, ursolic acid inhibited the migration and invasion of human CRC HCT116 and HCT-8 cells by interfering with the TGF-β1/ZEB1/miR-200c signaling network [221]. Compared with control mice, Modified Shenlingbaizhu Decoction (MSD) treatment significantly reduced the size of CRC tumors and the serum content of TGF-β1. Similarly, MSD inhibited CRC cell migration and invasion by limiting TGF-β/SMAD signaling [222]. Qingjie Fuzheng granule (QFG), a traditional Chinese medicine, suppressed the growth, wound-healing abilities and migration of HCT-8 and HCT116 cells. Moreover, QFG decreased the expression of lncRNA ANRIL, TGF-β1, p-SMAD2/3, SMAD4 and N-cadherin in CRC cells, suggesting that QFG inhibits the metastasis of CRC through the TGF-β1/SMAD axis [223]. Combined with the TCGA database results and previous network pharmacology, reports indicated that Fuzheng Xiaojijinzhan might play an anti-CRC metastasis role by inhibiting the TGF-β-Snail1 pathway [224].

Since strong genetic heterogeneity exists in CRC patients, development of personalized medicine for CRC patients is of extraordinary significance and value in clinical trials [225]. Based on large-scale data sharing and analytics, CRC is divided into four CMSs with distinguishing features: CMS1 (microsatellite instability immune, 14%), CMS2 (canonical, 37%), CMS3 (metabolic, 13%) and CMS4 (mesenchymal, 23%). Among them, CMS4 has prominent TGF-β activation, stromal invasion, angiogenesis and an immunosuppressive phenotype [226]. In the past decade, more and more efforts have been made to select the appropriate patient subsets for specific treatment of mCRC. Although the development of novel biological agents for therapies such as VEGF and EGFR has further changed the prospects for the treatments of mCRC, not all patients respond similarly to these therapies, so individualized medical treatments are in great need [227]. Designing personalized medicine targeting TGF-β signaling is definitely a good choice for CMS4 subtypes patients of CRC.

**Table 1:** Summary of factors targeting TGF-β signaling and acting on CRC metastasis.

Types		Targets	Involvements in Metastasis	Clinical Application	References
LncRNAs	MIR22HG	TGF-β pathway	Interact with SMAD2 and inhibit EMT	Facilitating immunotherapy in CRC	[187]
	MIR31HG	TGF-β pathway	Promote CRC cell migration and immunosuppression	Biomarker of cellular state	[181]
	EZR-AS1	TGF-β pathway	Promote CRC cell migration, proliferation and EMT	—	[186]
	TP73-AS1	TGF-β1	Promote CRC cell migration	Prognosis marker in CRC	[183]
	MIR503HG	TGF-β2	Inhibit CRC cell migration	Prognosis marker in CRC	[184]
	HOXC-AS3	TGF-β2	Reverse the effect of overexpression of TGF-β2	—	[185]
	CTBP1-AS2	TGF-β/SMAD2/3 pathway	Promote CRC cell migration and inhibit apoptosis	Prognosis marker in CRC	[182]
	LINC00941	TGF-β/SMAD2/3 pathway	Prevent SMAD4 protein degradation and activate EMT	Prognosis marker in CRC	[188]
miRNAs	miR-425	PTEN-P53/TGF-β	Inhibit cellular immune function	Shortened overall survival	[190]
	miR-576	PTEN-P53/TGF-β	Inhibit cellular immune function	Shortened overall survival	[190]
	miR-329	TGF-β1	Inhibit CRC cell migration	—	[191]
	miR-203	TGF-β2	Inhibit EMT	—	[193]
	miR-490-3p	TGFBR1	Inhibit CRC cell migration	Associated with poor prognosis of survival	[197]
	miR-301a	TGFBR2	Promote CRC cell migration	—	[194]
	miR-371~373	TGFBR2	Decrease tumor-initiating potential of CRC cells	—	[195]
	miR-3191	TGFBR2	Promote CRC cell migration	—	[196]
	miR-645	TGFBR2	Promote CRC cell migration and glycolysis	—	[198]
	miR-424	TGFBR3/SMAD7	Promote CRC cell migration and arrest cell cycle/promote proliferation	—	[199,206]
	miR-27a	SMAD2 and SGPP1	Inhibit CRC cell migration and promote apoptosis	Biomarker for monitoring CRC development and progression	[200]
	miR-140	SMAD3	Inhibit CRC cell migration	—	[201]
	miR-20a-5p	SMAD4	Promote CRC cell migration and EMT	Predicts poor prognosis in CRC patients	[202]
	miR-25	SMAD7	Inhibit CRC cell migration	—	[203]
	miR-4775	SMAD7	Promote CRC cell migration and EMT	Predicts poor survival	[204]
	miR-21	SMAD7	Accelerate TGF-β dependent EMT	—	[205]
circRNAs	circFAM120B	miR-645	Inhibit CRC cell migration and glycolysis	—	[198]
	circTBL1XR1	miR-424	Promote CRC cell migration	—	[206]
Kinase Inhibitors	Sitagliptin	TGF-β1	Inhibit EMT and impair cell cycle	Prevents colon cancer and lung metastasis in animal models and humans	[210]
	LY2157299	TGF-β receptor	Mitigate TGF-β driven impairment of NK cell cytotoxicity	Currently in clinical trials for various malignancies	[209]
	LY2109761	TGF-β receptor	Downregulated the phosphorylation of SMAD2	Applied mostly in preclinical animal experiments	[208]
	regorafenib	TGF-β/SMAD4 pathway	—	While using regorafenib, patients with SMAD4 mutation or activation of TGF-β pathway showed a worse PFS	[169]
Natural com-pounds and Chinese herbal formulas	solasodine	TGF-β1	Inhibit CRC cell stemness and EMT	—	[215]
	MSD	TGF-β1	Inhibit CRC cell migration	Reduced the size of CRC tumors in mouse model	[222]
	Celastrol	TGF-β1/SMAD pathway	Inhibit CRC cell migration	—	[218]
	baicalin	TGF-β/SMAD pathway	Inhibit EMT, stemness and cell cycle	—	[217]
	QFG	TGF-β/SMAD pathway	Inhibit CRC cells growth and migration	—	[223]
	*Hedyotis diffusa* Willd	TGF-β/SMAD4 pathway	Inhibit CRC cell migration and EMT	—	[216]
	ursolic acid	TGF-β1/ZEB1/ miR-200c	Inhibit CRC cell migration	—	[221]
	metformin	TGF-β ligand and PI3K/AKT pathway	Arrest cell cycle and inhibit cell proliferation	—	[220]
	EESB	PI3K/AKT and TGF-β/SMAD signaling	Inhibit CRC cell migration and decrease the expression of MMPs	—	[219]
	Fuzheng Xiaojijinzhan	TGF-β-Snail1	Anti-CRC metastasis role	—	[224]

## 6. Conclusions and Future Perspective

Metastatic CRC is an intractable disease due to its poor prognosis, high mortality and limited optimal therapies in clinical situations worldwide, even in developed countries. Several key areas in mCRC research include: early identification of metastasis, recognition of specific prognostic and predictive biomarkers, discovery of new molecular targets, development of new drugs and clinical operations. TGF-β represents a conserved signaling pathway that is widely involved in various physiological and pathological processes. In this review, we summarized the changes in the TGF-β signaling pathway in mCRC patients, its functional mechanisms and its possible applications in mCRC diagnosis, prognosis and potential targeted therapies in clinical trials. We explained in detail that TGF-β signaling functions to promote EMT, facilitate angiogenesis, suppress anti-tumor activity of the immune cells in the microenvironment and contribute to stemness of CRC cells in mCRC (as shown in Figure 1). Following these working mechanisms of TGF-β signaling in mCRC, molecular targeting therapies aimed at the different key factors upstream and downstream of TGF-β signaling could be accordingly developed to improve the efficacy and safety of treatments, especially in MCS4 subtypes of mCRC.

Various remaining problems regarding TGF-β signaling in CRC metastasis still need to be clarified. Initially, TGF-β was not considered as a good target for tumor treatments because of its dual roles in tumor early development and late-stage metastasis. However, recent years witnessed an increasing number of new therapies that target TGF-β signaling in CRC metastasis. These include suppressing TGF-β or downstream components of the signaling, blocking crosstalk between TGF-β signaling pathways and other signal pathways and redirecting TGF-β signaling from pro-tumor to anti-tumor functions in CRC metastasis [228]. Additionally, because mCRC is a complicated disease, we should attach importance to not only TGF-β signals from inside but also outside of CRC cells, namely from the stroma cells and microenvironment of mCRC. Hopefully, in-depth and systematic studies in this research field will help us understand more in the future.

Due to the heterogeneity of CRC cells and individual differences among mCRC patients, it is impossible to find an optimal treatment strategy that fits everyone. Thanks to improved knowledge of the molecular mechanisms underlying CRC metastasis, promising advances help us modify traditional treatments. Recent reports demonstrated that targeting TGF-β could be combined with other signal inhibitors such as combinatorial synergy, reverse therapy resistance or sensitize radiotherapy to achieve a sustained therapy response in CRC patients [28,229]. Moreover, since TGF-β signaling is an immunosuppressive regulator in the TME of mCRC, there is great potentials in combining TGF-β targeting with immunotherapy agents to enhance the efficacy and benefit for patients. In order to move forward, applications of NGS and genetic profiling in clinical trials will help us characterize the molecular subtypes of TGF-β signaling in mCRC patients, and appropriate personalized medicine specifically targeting TGF-β can be definitely and smoothly translated into mCRC treatments.

## Abbreviations

5-FU	5-fluorouracil
αSMA	alpha smooth muscle actin
ACSL3	acyl-CoA synthetases 3
ACVR2A	activin A receptor type 2A
AKT	protein kinase B
APC	adenomatous polyposis coli
aPKC-ι	atypical protein kinase C-ι
ASE	allele-specific expression
ASS1	argininosuccinate synthase 1
AT1a	angiotensin II subtype receptor 1a
BMP	bone morphogenetic protein
CAFs	cancer-associated fibroblasts
CCBE1	collagen and calcium-binding EGF domain-1
CEA	Carcinoembryonic antigen
CMS	consensus molecular subtype
Co-SMAD	common-mediator SMAD
CRC	colorectal cancer
CRLM	colorectal liver metastases
CSCs	cancer stem cells
Cten	C-terminal tensin-like
CTHRC1	collagen triple helix repeat containing 1
CXCL12	C-X-C motif chemokine ligand 12
CXCR4	C-X-C motif chemokine receptor type 4
DCs	dendritic cells
ECM	extracellular matrix
EESB	ethanol extract of Scutellaria barbata D. Don
EGFR	epidermal growth factor receptor
EMT	epithelial-mesenchymal transition
EO	early onset
FFPE	formalin-fixed paraffin-embedded
Foxp3	forkhead box protein 3
GDF	growth and differentiation factor
GPC1	Glypicans 1
GREM1	gremlin 1
HAPLN1	hyaluronan and proteoglycan link protein-1
hCGβ	beta human chorionic gonadotropin
HLTF	helicase-like transcription factor
IL-11	interleukin-11
INHBA	inhibin, beta A
INHBB	inhibin subunit beta B
ISLR	immunoglobulin superfamily containing leucine-rich repeat
I-SMAD	inhibitory SMAD
KEGG	Kyoto Encyclopedia of Genes and Genomes
lncRNA-ATB	lncRNA-activated by TGF-β
lncRNA EZR-AS1	lncRNA ezrin antisense RNA 1
lncRNAs	long non-coding RNAs
MAPK	mitogen-activated protein kinase
mCRC	metastatic colorectal cancer
MiR	microRMA
MMPs	matrix metalloproteinases
MSD	Modified Shenlingbaizhu Decoction
MSI	microsatellite instability
NEP	neutral endopeptidase
NF-κB	nuclear factor-κB
NGS	next-generation sequencing
OS	overall survival
PARG	poly (ADP-ribose) glycohydrolase
PDGF-D	platelet-derived growth factor-D
PD-L1	programmed cell death-Ligand 1
PFS	progression-free survival
P4HA3	prolyl 4-hydroxylase subunit alpha 3
PI3K	phosphoinositide 3-kinase
PNMA5	Paraneoplastic antigen Ma family number 5
PODXL	podocalyxin-like
QFG	Qingjie Fuzheng granule
RGC-32	response gene to complement 32
ROCK	Rho-associated kinase
R-SMAD	receptor-regulated SMAD
RUNX	runt related transcription factor
SDF-1	stromal cell derived factor-1
SNP	single nucleotide polymorphism
STAT3	signal transducer and activator of transcription 3
TAK1	TGF-β-activated kinase 1
TAMs	tumor-associated macrophages
TANs	tumor-associated neutrophils
TCGA	The Cancer Genome Atlas
TGF-β	transforming growth factor-β
TGFBI	TGF-β-induced protein ig-h3
TH1	type 1 T-helper cell
Th17	T helper 17
THBS4	thrombospondin-4
THSD4	thrombospondin type-1 domain-containing protein 4
TME	tumor microenvironment
TMEM45A	transmembrane protein 45A
TRAIL	tumor necrosis factor-related apoptosis-inducing ligand
Tregs	regulatory T cells
TRIM25	tripartite motif containing 25
Trp53	transformation related protein 53
TSIPs	tumor spheres with inverted polarity
TSP1	thrombospondin 1
VEGF	vascular endothelial growth factor
